# Temporal Patterns of Engagement and Sentiment in a Suicide Prevention Mobile App: Three-Year Observational Study

**DOI:** 10.2196/95374

**Published:** 2026-07-16

**Authors:** Elia Gabarron, Nathan Massicot, Kerstin Denecke

**Affiliations:** 1 Polibienestar Research Institute, Universitat de València, València, Spain; 2 Institute for Patient-centered Digital Health, Department of Engineering and Computer Science, Bern University of Applied Sciences, Quellgasse 21, Biel, Bern, 2502, Switzerland, 41 32 321 67 94

**Keywords:** suicide prevention, mobile app, digital mental health, temporal patterns, engagement, sentiment analysis, circadian rhythms

## Abstract

**Background:**

Temporal fluctuations in distress and suicidal ideation across daily, weekly, and seasonal cycles may influence the use and effectiveness of digital suicide prevention tools. Understanding patterns of app engagement, perceived suffering, and affective expression can inform the design of proactive, personalized digital interventions, thereby impacting adherence and efficacy.

**Objective:**

This study aimed to examine temporal patterns of engagement with 2 core components of the suicide prevention app SERO (Suicide Prevention: a Uniform Effort, Resource-Oriented; BFH, Lucerne Psychiatry), specifically the safety plan and the PRISM-S (Pictorial Representation of Illness and Self-Measure—Suicidality) self-assessment, using 3 years of interaction log data, assessing variations across circadian, weekly, and seasonal cycles, and evaluating the sentiment of free-text responses submitted immediately after PRISM-S self-assessments.

**Methods:**

We analyzed anonymized interaction logs from the SERO app collected over 3 years (November 2022 to December 2025). Engagement metrics included the frequency of use of the safety planning functionality and PRISM-S self-assessment entries. Free-text responses provided after PRISM-S assessments were analyzed using automated sentiment classification. Temporal analyses examined variations by the hour of the day, day of the week, and season. One-way ANOVAs, post hoc tests, and Pearson correlations were used to examine patterns and associations between perceived suffering and sentiment.

**Results:**

A total of 1076 users engaged with the safety planning functionality of the SERO app, generating 3502 entries, with coping strategies and warning signs showing the highest mean interactions and personal beliefs the lowest. Separately, 1212 app users accessed the PRISM-S self-assessment, producing 2329 entries (mean distance 12.91, 95% CI 12.39‐13.42 cm), with most app users recording only 1 or 2 registrations. Safety planning engagement showed clear diurnal patterns, peaking in the afternoon (2 PM to 3 PM) and being lowest at night (midnight to 3 AM), whereas PRISM-S scores were stable across time. Sentiment analysis revealed predominantly negative affect (mean score of −0.41, SD 0.51, 95% CI −0.44 to −0.39), correlated with PRISM-S distance, and was most negative at night (specifically at 11 PM) and during the afternoon (2 PM to 5 PM). Seasonal effects were small but significant for PRISM-S, with the lowest perceived suffering in summer.

**Conclusions:**

Digital suicide prevention tools can support routine patterns of coping behavior, but periods of increased reported distress, particularly at night, may be underaddressed. Integrating automated sentiment analysis alongside self-assessments could potentially enable personalized, time-adaptive interventions that detect changes in emotional state and deliver timely, tailored support, thereby strengthening proactive engagement and resilience.

## Introduction

### Temporal Patterns in Mental Health and Suicidal Behavior

Temporal patterns refer to systematic, predictable fluctuations in human behavior, symptoms, or events across distinct time scales, including circadian rhythms (daily, 24-hour cycles), circaseptan rhythms (weekly, 7-day cycles), and circannual rhythms (seasonal, yearly cycles). In mental health, these patterns are highly relevant because they reflect periods of elevated distress or vulnerability. Observational studies among university populations have shown that self-reported mood and well-being are consistently better in the morning, often peaking upon waking, and worse late at night, particularly around midnight [1,2]. However, findings have not been entirely consistent across studies. For example, research conducted with healthy adults has shown that mood and well-being may instead reach their highest levels during the circadian evening [3], whereas another study found no significant diurnal fluctuations in overall mood valence; however, levels of calmness increased progressively throughout the day [4].

Research indicates that sleep-circadian disruptions play a key transdiagnostic role across a range of psychiatric disorders [5], highlighting the potential value of incorporating circadian factors into clinical interventions [6-8], particularly those aimed at preventing suicide. This is supported by evidence showing that measures of distress and suicidality in everyday life show regular, predictable changes across hours, days, and seasons, suggesting that interventions timed to these patterns could more effectively target periods of heightened distress or need for support, which may not necessarily coincide with the timing of suicidal behavior [9]. Research has shown that self-harm presentations most often occur outside standard working hours (9 AM to 5 PM, Monday to Friday), with peaks frequently observed in the hours just before and after midnight [10]. Also, online behaviors seem to mirror these trends, with searches and postings for terms such as “suicide” and “depression” typically peaking during the late evening or night [11,12]. Analysis of Reddit’s SuicideWatch forum has also shown clear diurnal variation, with the highest proportion of posts occurring in the early morning (2 AM to 5 AM) and a trough in the late morning or early afternoon [13]. These diurnal patterns are further influenced by the day of the week and season, with mood and well-being generally reported as better on weekdays and in summer compared to winter [2].

Studies have also shown circaseptan (weekly) patterns in both behavior and suicide mortality. Multiple multicountry analyses have indicated that suicide deaths consistently vary across the week, with Mondays showing the highest risk and weekends generally the lowest, although elevated weekend rates have been reported in parts of South and Central America, Finland, and South Africa [14-16]. Research on youth in the United States (aged 10‐18 y) has identified peaks in suicide deaths on Mondays, Tuesdays, and Wednesdays, as well as during March, April, and October [17]. Weekly fluctuations have also been observed in online and social media behavior, with searches and posts frequently increasing on weekends [12] or on specific weekdays [13], depending on the population and platform studied.

There is also evidence of pronounced seasonal and holiday-related patterns in suicide and well-being. Suicide mortality often peaks in spring [15,18-20] or summer [15,20,21], with notable troughs around holidays such as Christmas and marked increases on New Year’s Day, as well as during paydays or other specific periods [14,18,21,22]. Consistent with these findings, research on self-reported well-being has shown higher mood levels in summer compared to winter [2], highlighting the impact of environmental and social cycles on mood and suicidal behavior.

These temporal fluctuations could indicate periods of heightened vulnerability during which app users are more likely to seek support, potentially reflecting shifts in distress levels or coping resources across the day, week, or year. However, such time-dependent patterns remain underexplored in the context of digital mental health tools, particularly suicide prevention apps. In addition to temporal patterns of engagement, textual expressions generated by app users may provide further insights into their emotional state and perceived level of distress. Previous studies have shown that sentiment analysis can identify fluctuations in affect and mental health indicators in online communication, including suicidal ideation [23,24]. In the context of digital mental health tools, analyzing app users’ free-text inputs may therefore offer complementary information about perceived distress, proximity to suicidality, and the emotional experiences that accompany app engagement.

### Objective

The objective of this study is to examine temporal patterns of engagement with 2 core components of the suicide prevention app SERO (Suicide Prevention: a Uniform Effort, Resource-Oriented), specifically the safety plan and the PRISM-S (Pictorial Representation of Illness and Self-Measure—Suicidality) self-assessment, using 3 years of interaction log data, assessing variations across circadian, weekly, and seasonal cycles, and evaluating the sentiment of free-text responses submitted immediately after PRISM-S self-assessments.

## Methods

### Study Design

In this study, we analyzed data reflecting app users’ engagement with the safety plan and the PRISM-S self-assessment embedded in the SERO app during the period from November 24, 2022, to December 19, 2025, following the STROBE (Strengthening the Reporting of Observational Studies in Epidemiology) reporting guidelines for observational studies (Checklist 1) [25].

### SERO App

SERO is a self-management mobile app for suicide prevention, designed to support people experiencing suicidal thoughts or behaviors, as well as their trusted contacts, before, during, and after suicidal crises [26]. It was developed in collaboration with individuals at risk, their relatives, and health care professionals. The app offers self-management support through an individual safety plan, a visual self-assessment of perceived suffering with reflection questions, a treasure chest, and information on crisis hotlines. The app has been available on iOS and Android since November 2022 and is offered in German, French, Italian, and English. Luzerner Psychiatrie AG is the responsible party, and Bern University of Applied Sciences is responsible for its maintenance and development. App users register with the health data bank MIDATA, a General Data Protection Regulation–compliant health data storage. All data entered into the SERO app is stored at MIDATA. Additionally, all app users provide consent during registration for the analysis of their data for research purposes.

The app helps users prepare actionable steps for moments of crisis and emergencies through an individual safety plan functionality that can be shared with trusted persons. Designed to support app users in moments of distress, the safety plan functionality aligns with research showing that both in-person and digital safety planning interventions can improve suicidal thoughts and behaviors [27-29]. This feature consists of 5 parts: coping strategies, warning signs, motivation, distraction strategies, and personal beliefs. App users may complete these sections voluntarily, edit or update the content at any time, or leave any section blank according to their preferences. This flexible design allows individuals to tailor the safety plan to their personal needs and circumstances, thereby supporting autonomy and user-centered engagement [26].

The app is complemented by the subjective self-assessment tool for suicidality, PRISM-S [30,31]. PRISM-S is a very brief visual assessment tool. It shows strong convergent validity with tools like the Beck Scale for Suicide Ideation (*r*≈−0.73) and the Depressive Symptom Inventory—Suicidality Subscale (*r*=−0.59 to −0.76) [30,31]. This visual tool asks a person to place a marker representing suicidal thoughts in relation to a symbol of the self. PRISM-S can be used to assess how far an individual at risk is literally “away” from a suicidal act. The measured distance between the 2 markers (ranging from 0=“close to crisis” to 25=“feeling safe”) reflects perceived suffering: shorter distances indicate that suicidality feels close and intrusive, whereas longer distances suggest greater psychological separation and a reduced perceived impact. PRISM-S does not have established cutoff scores or fixed thresholds; rather, it is intended as a continuous, subjective indicator of perceived proximity and burden. However, like other self-report instruments, it should be interpreted as a subjective, momentary indicator of perceived suffering and proximity to suicidality, primarily reflecting current perceived distress rather than providing an objective measure of suicide risk or predicting future suicidal behavior. The visual PRISM-S assessment is complemented by a 5-question survey covering personal interpretation, thoughts, physical perceptions, emotions, and resilience. These questions can be answered with free-text responses.

The app also provides crisis hotline numbers, quick access to personal emergency contacts, and curated links to further support services. In addition, SERO includes a “Treasure Chest” feature, where app users can store photos, supportive memories, or coping strategies (text, images, and links) to use as anchors during difficult moments.

A second key user group of the app consists of relatives and trusted persons, who can access the safety plan of an individual at risk when it has been shared with them, fill out a resource plan for self-care, and get information to better support the person at risk. The app is explicitly positioned by the responsible party within a broader support network, involving health professionals and crisis services (eg, hotline numbers and links to counseling offers).

### Data Analysis

We base our analysis on the usage data from the SERO app since its release in 2022, specifically from November 24, 2022, to December 19, 2025. The anonymized data contain app usage behavior. Furthermore, we analyzed the free-text responses given after a PRISM-S assessment was conducted. No additional data entered by the user in the app were analyzed for this paper. The content of the safety plans had already been analyzed and described in previous work [32].

Data were analyzed using IBM SPSS [version 28.0.1.1 (14)]. Descriptive statistics were used to summarize user characteristics and engagement with the PRISM-S and safety planning functionalities, including frequencies, percentages, and total counts of registrations per user. Aggregation procedures were applied to calculate the total number of entries per user and to identify patterns of repeated use throughout the study period. All dates were classified into meteorological seasons (winter: December to February, spring: March to May, summer: June to August, and autumn: September to November). Hours were classified into 3 blocks (morning: 6 AM to 1:59 PM, afternoon or evening: 2 PM to 9:59 PM, and night: 10 PM to 5:59 AM). To analyze the data, each time a user accessed any app functionality, the date and hour of access were considered, and sessions with no further interaction were treated as having zero interactions. Analyses were conducted at the aggregate level of app interactions and do not account for within-user longitudinal variability due to the anonymized structure of the dataset. To examine temporal variations in safety planning and PRISM-S recordings, one-way ANOVAs were conducted to test for differences in recorded distances across days of the week, time blocks within the day, and seasons of the year.

The analyses in this study were primarily exploratory and aimed at identifying temporal patterns in real-world app usage across multiple time scales (hour of the day, weekday, and season). Given this exploratory framework and the absence of a single confirmatory hypothesis, we did not apply a global correction for multiple testing across all analyses. Instead, results were interpreted jointly using *P* values, effect sizes, and CIs to reduce overreliance on statistical significance alone. Post hoc pairwise comparisons were conducted using Tukey honestly significant difference (HSD) or Bonferroni adjustments, where appropriate, within specific ANOVA models.

Free-text responses provided immediately after a PRISM-S self-assessment were analyzed to quantify sentiment and examine their association with the PRISM-S distance metric. Text entries were first cleaned by whitespace normalization only; no stop-word removal, lemmatization, or translation was applied in order to preserve the affective cues (negation, intensifiers, and code-mixing across languages) on which transformer-based classifiers depend. For each questionnaire submission (1 user × 1 timestamp), the answers to the 5 reflection questions were concatenated into a single text and treated jointly. Each aggregated submission was then linked to the corresponding PRISM-S distance using an exact inner join on the user identifier and timestamp. Of the 2329 PRISM-S entries, 1286 had a matching free-text submission at the same timestamp and were retained for sentiment analysis; 50 text submissions had no matching distance, and 662 PRISM-S entries had no accompanying text.

Sentiment was estimated in Python using the multilingual transformer model cardiffnlp/twitter-xlm-roberta-base-sentiment (Hugging Face Transformers; max sequence length of 512 tokens, CPU inference) [33]. This model is an XLM-RoBERTa-base architecture pretrained on approximately 198 million tweets in more than 30 languages and fine-tuned for 3-way sentiment classification (negative, neutral, and positive) on a multilingual benchmark that covers the 4 languages supported by the SERO app: German, French, Italian, and English. We selected this model because the free-text inputs in SERO share key properties with social media microposts on which it was trained: short length, an informal first-person register, frequent code-mixing across the 4 app languages, and emotionally loaded content describing feelings and inner states. Compared with general-purpose multilingual sentiment classifiers fine-tuned on long, formal text (eg, product reviews, news, and clinical narratives), the Twitter–fine-tuned multilingual variant offered the closest match to our input distribution. We acknowledge that no sentiment model has been formally validated on suicide prevention–related free text, and the score is, therefore, interpreted as a relative indicator of affective tone rather than a clinically validated risk metric.

For each submission, the model returned softmax probabilities: P(negative), P(neutral), and P(positive). We summarized these probabilities as a continuous polarity score defined as follows: score = P(positive) − P(negative), bounded within [−1,+1] and requiring no tuning constants or post hoc adjustments. Submissions with scores <−0.20 were classified as negative, scores between −0.20 and +0.20 as neutral, and scores ≥+0.20 as positive. To test whether sentiment was related to perceived crisis proximity, we computed a Pearson correlation between PRISM-S distance and the sentiment score (Pearson *r*=0.506; *P*<.001) and fitted an ordinary least-squares linear regression to summarize the direction and strength of the relationship. Differences in mean sentiment across hours of the day, days of the week, and meteorological seasons were tested with one-way ANOVA, complemented by the nonparametric Kruskal-Wallis *H* test as a robustness check.

To assess whether the choice of score formulation influenced the reported associations, we replicated the analysis using 4 alternative score definitions derived from the same model probabilities (argmax label, strongest signed polarity, and 2 combinations of strongest polarity with the polarity margin damped by the neutral probability). Across all definitions, the Pearson correlation with PRISM-S distance ranged between *r*=0.46 and *r*=0.51 (all *P*<.001), and the qualitative findings—sign of the correlation, significant variation across hours, nonsignificant variation across weekdays, a significant seasonal effect, and the autumn or winter ranking—were preserved. The predominantly negative classification of submissions was also robust across cutoffs from ±0.10 to ±0.33 (negative class: 67.9%-74.7%).

### Ethical Considerations

User involvement in the SERO project was approved by the ethics committee of Northwestern and Central Switzerland under number 2022‐00870. All app users provided consent to the anonymized data usage for research purposes when registering. The privacy policy regulates the use of the data. Participants did not receive any compensation.

## Results

### Description of the Sample

Regarding the data collected through the SERO app, a total of 1076 users engaged with the safety planning functionality, and 1182 accessed the PRISM-S functionality. The 1076 app users who engaged with the safety plan functionality registered 3502 entries within the safety planning options. Across topics, app users engaged an average of 4.06 times with each of the 5 topics of the safety plan. The highest mean number of interactions per user with safety plan elements was observed for coping strategies (mean 4.43, 95% CI 4.19-4.66; range 1‐24; 776/3502) and warning signs (mean 4.38, 95% CI 4.14-4.62; range 1‐22; 778/3502), followed by motivation (mean 4.19, 95% CI 3.96-4.43; range 1‐21; 707/3502) and distraction strategies (mean 4.04, 95% CI 3.81-4.27; range 1‐31; 705/3502). Personal beliefs showed the lowest mean number of interactions per user (mean 2.90, 95% CI 2.69-3.10; range 1‐21; 536/3502).

Additionally, 1212 individual app users accessed the PRISM-S functionality, generating 2329 PRISM-S entries. Most app users registered PRISM-S values only once (850/2329, 36.5%), followed by 2 registrations (342/2329, 14.7%) and 3 registrations (216/2329, 9.3%). Overall, 63.5% (770/1212) of app users recorded 1 or 2 entries, indicating that repeated use was less frequent, with progressively smaller proportions of users contributing higher numbers of registrations. A total of 30 app users entered the PRISM-S functionality, but they did not register any values. The mean distance was 12.905 cm (95% CI 12.392‐13.418), with values ranging from 0 to 24.9 cm.

The first interaction with the safety plan occurred on November 30, 2022, and the most recent on December 18, 2025. For the PRISM-S assessment, the earliest recorded use was on December 5, 2022, with the latest on December 17, 2025.

### Temporal Patterns of Engagement With the Safety Plan and the PRISM-S Self-Assessment Functionality

#### Hours of the Day

**Figure 1.**
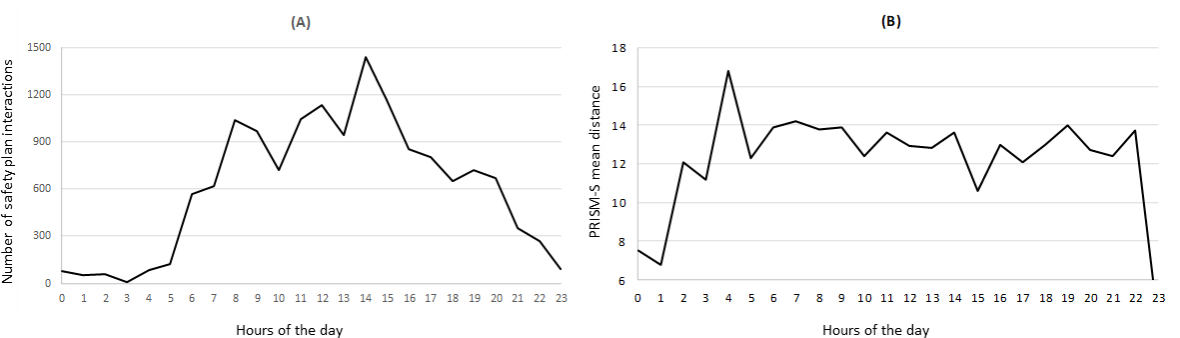
(A) Safety plan interactions and (B) mean PRISM-S (Pictorial Representation of Illness Self-Measure—Suicidality) distance scores across hours of the day.

The number of interactions with the safety plan varied substantially across hours of the day, with interactions being lowest during the early morning (midnight to 3 AM; means ranging from 5.00, 95% CI 5-5 to 21.85, 95% CI 19.97-23.73) and increasing throughout the day to peak in the afternoon (2 PM to 3 PM; mean 24.18, 95% CI 23.42-24.95, and 18.45, 95% CI 17.89-19.01, respectively), before gradually declining in the evening and late at night. The one-way ANOVA indicated significant differences in the mean number of interactions across hours (*F*_23,14401_=1157.43; *P*<.001). The Kruskal-Wallis test confirmed significant differences in the distribution of interactions across hours (*H*_23_=8832.43; *P*<.001), with the highest activity observed between noon and 4 PM and the lowest activity during early morning and late-night hours (Figure 1A).

The one-way ANOVA was conducted to examine whether distance varied across different hours of the day. Post hoc comparisons using the Tukey HSD test indicated that there were no statistically significant differences in the mean distance between any pair of hours (all *P*>.05; Figure 1B).

The number of interactions with the safety plan differed significantly across time blocks (*F*_2,14422_=1720.44; *P*<.001). The mean number of interactions was lowest during the night (mean 33.44, 95% CI 31.98‐34.89) and substantially higher during the morning (mean 191.45, 95% CI 190.09‐192.82) and afternoon or evening (mean 196.36, 95% CI 194.20‐198.51). The Tukey HSD test indicated that interactions during the night were significantly lower than during both the morning and afternoon or evening periods (all *P*<.001). The difference between morning and afternoon or evening was small but also statistically significant (mean difference 4.91; *P*<.001; Multimedia Appendix 1).

Descriptive analyses indicated that the mean distance in PRISM-S varied slightly across time blocks, with the highest average observed in the morning (mean 13.41, 95% CI 12.655‐14.162), followed by the afternoon or evening (mean 12.63, 95% CI 11.880‐13.383), and the lowest at night (mean 11.27, 95% CI 9.367‐13.220; Multimedia Appendix 1). A one-way ANOVA showed no statistically significant differences between time blocks (*F*_2,1179_=2.57; *P*=.08), with a very small effect size (η²=0.004). Post hoc comparisons using Tukey HSD indicated that none of the pairwise differences reached significance.

#### Days of the Week

Mean daily interactions with the safety plan ranged from 3.71 (95% CI 3.49-3.94) on Wednesday to 4.18 (95% CI 3.93-4.44) on Thursday. Thursday and Saturday showed slightly higher mean interactions (4.18, 95% CI 3.93-4.44 and 4.16, 95% CI 3.81–4.51, respectively). A one-way ANOVA showed no statistically significant differences in interactions across weekdays (*F*_6,3609_=1.91; *P*=.08). The analysis of intersubject factors showed that the number of interactions varied across topics but not significantly across days of the week. Thursday had the highest number of interactions (n=686), followed by Tuesday (n=592) and Wednesday (n=555), whereas Saturday and Sunday had the lowest (n=324 and 394, respectively), but the effect of the day of the week (*F*_6,3534_=1.870; *P*=.08) and the interaction between day and topic (*F*_24,3534_=0.491; *P*=.98) were not significant. Post hoc Bonferroni comparisons showed that “personal beliefs” differed significantly from all other topics (*P*<.001), whereas differences among the other topics were mostly nonsignificant (Multimedia Appendix 2).

PRISM-S mean distances were also stable across the days of the week, ranging from 11.83 (Sunday) to 13.13 (Thursday; Multimedia Appendix 2). The one-way ANOVA showed no statistically significant differences in PRISM-S distance between the days (*F*_6,2286_=1.33; *P*=.24). Effect sizes were negligible (η²=0.003, 95% CI 0.000‐0.007; ω²≈0.001).

#### Seasons

**Figure 2.**
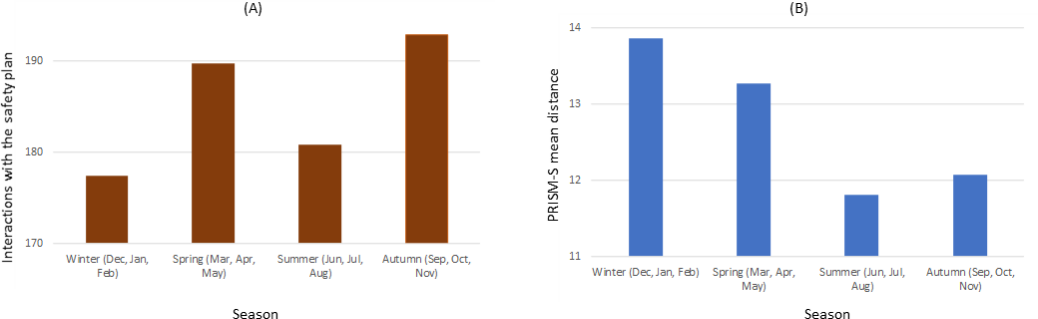
(A) Interactions with the safety plan functionality across seasons. (B) Mean Pictorial Representation of Illness Self-Measure—Suicidality (PRISM-S) distance across seasons.

The number of interactions with the safety planning functionality varied slightly across seasons, with the lowest average in winter (mean 177.35, 95% CI 174.77‐179.94) and the highest in autumn (mean 192.80, 95% CI 190.05‐195.54). Spring and summer presented intermediate values (mean 189.68, 95% CI 187.03‐192.33 and mean 180.77, 95% CI 178.18‐183.37, respectively). Post hoc comparisons using Tukey HSD indicated that none of the pairwise differences between seasons were statistically significant (all *P*>.28). Levene tests indicated significant heterogeneity of variances across groups (*P*<.001; Figure 2A).

The mean PRISM-S distances varied across seasons, with the highest average observed in winter (December to February; mean 13.866, 95% CI 12.887-14.845), followed by spring (March to May; mean 13.277, 95% CI 12.343‐14.211), autumn (September to November; mean 12.067, 95% CI 10.995‐13.139), and summer (June to August; mean 11.807, 95% CI 10.649‐12.965; Figure 2B). The Levene test indicated homogeneity of variances (*P*>.05), supporting the use of ANOVA. The one-way ANOVA showed a significant effect of season on distance (*F*_3,1178_=3.31; *P*=.02). Post hoc Tukey comparisons showed that PRISM-S self-reported distances in summer were significantly lower than in winter (mean difference 2.06; *P*=.045), while other pairwise comparisons were not statistically significant. Complementary regression analyses using sine and cosine transformations of month confirmed a small but significant cyclic pattern in distance (*R*²=0.011; *F*_2,1179_=6.81; *P*=.001).

### Sentiment Patterns in Relation to Crisis Proximity (PRISM-S) and Time

**Figure 3.**
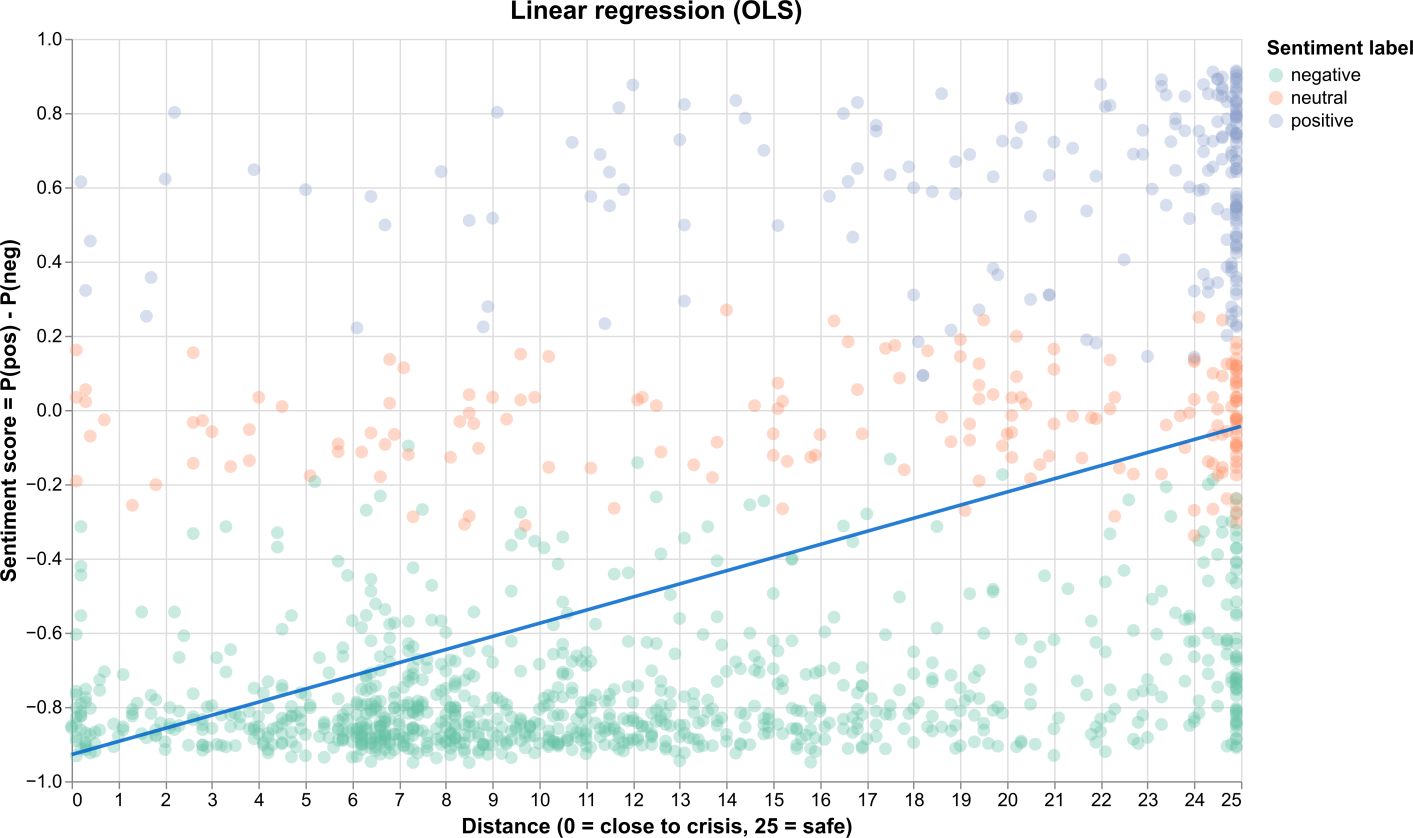
Correlation between the Pictorial Representation of Illness Self-Measure—Suicidality (PRISM-S) distance and sentiment. OLS: ordinary least squares.

Sentiment scores (ranging from −1=“negative” to +1=“positive”) were negatively skewed across the full sample (mean −0.448, SD 0.547), indicating a predominantly negative affective tone. Sentiment showed a statistically significant positive correlation with PRISM-S crisis distance (Pearson *r*=0.506; *P*<.001), in the sense that greater proximity to crisis was associated with more negative sentiment (Figure 3). Analyses by time of day revealed significant variation in mean sentiment across hours (ANOVA: *F*_23,1262_=1.79; *P*=.01; Kruskal-Wallis *H*=45.30; *P*=.004), with the most negative scores observed during night hours (particularly 11 PM and 2 AM to 5 AM). No significant differences in sentiment were found across days of the week (ANOVA: *P*=.62). Seasonal analysis indicated significant variation by season (ANOVA: *F*_3,1282_=4.64; *P*=.003), with autumn being the season with the most negative mean sentiment (−0.544) and winter the least negative (−0.376), though this pattern was not significant under the nonparametric test (Kruskal-Wallis: *P*=.08).

## Discussion

### Summary of Main Findings

A total of 1076 app users engaged with the safety planning functionality of the SERO app, generating 3502 entries across 5 topics, with coping strategies and warning signs showing the highest mean interactions per user, and personal beliefs showing the lowest. Separately, 1212 app users accessed the PRISM-S self-assessment, producing 2329 entries, with most app users recording only 1 or 2 registrations. The mean PRISM-S distances were 12.91 (95% CI 12.39‐13.42) cm.

Temporal analyses showed significant diurnal patterns in safety planning interactions: engagement was lowest during the early morning (midnight to 3 AM) and peaked in the afternoon (2 PM to 3 PM; *P*<.001), while interactions during the night were significantly lower than in the morning, afternoon, or evening periods. In contrast, PRISM-S scores were stable across hours of the day and weekdays, with negligible effect sizes. Seasonal analyses showed a small but significant effect for PRISM-S distances, with the lowest values in summer and the highest in winter (*P*=.02), whereas safety planning use did not vary significantly across seasons. These results suggest that while PRISM-S reflects relatively stable self-reported proximity to suicidality, safety planning engagement is time-of-day dependent. However, these patterns should be interpreted as descriptive associations rather than evidence of underlying temporal changes in risk.

Sentiment analysis of free-text responses from app users showed a predominantly negative affective tone (mean −0.41, SD 0.51, 95% CI −0.44 to −0.39) and was significantly associated with PRISM-S crisis proximity, with more negative sentiment observed when perceived suffering was lower. Sentiment also varied across the time of day, with the most negative expressions occurring during nighttime hours, especially at 11 PM, and from 2 AM to 5 AM.

### Diurnal Variations

The analysis of app users’ interactions with the SERO app showed some temporal usage patterns. Engagement with the safety planning functionality followed a diurnal rhythm: interactions were lowest between midnight and 3 AM, increased steadily across the day, peaked in the afternoon (2 PM to 3 PM), and then gradually declined in the evening. This pattern may reflect the cognitively demanding nature of filling out a safety plan, which depends on executive functions that are stronger during the daytime, when alertness and cognitive performance peak [34], thereby making app users more able to engage proactively with the tool’s functionality. The observed daily pattern may also mirror the typical circadian fluctuations of alertness, as described in technology usage patterns, with low activity at night, increasing after waking, peaking in the afternoon, and declining in the evening [35,36].

PRISM-S self-assessments of app users did not show statistically significant diurnal changes, but the morning-to-night decline in perceived suffering remains concerning and may reflect broader evidence that distress and acute risk indicators often peak at night [1,2,10-13]. Complementing these self-ratings, the sentiment analysis of free-text responses from app users, provided immediately after the PRISM-S self-assessment, showed a statistically significant correlation between measurements, suggesting that spontaneous free text may carry signals about the user’s internal state, consistent with previous research [37]. Particularly, the most negative sentiment scores were observed during nighttime hours, supporting the notion that emotional distress and suicidal ideation tend to intensify at night [1,2,10-13].

These findings suggest that app users tend to engage with safety planning during the day, while perceived suffering and expressed sentiment decline at night, creating a critical period when the preventive tool may be underused despite potentially elevated distress, which does not necessarily correspond to the timing of suicidal behavior [9]. This may point to a potential “window of opportunity” for digital suicide prevention, in which encouraging app use during periods of higher alertness could strengthen resilience and coping, positioning the tool as a means of proactive support rather than solely responding to immediate crises. Integrating automated sentiment analysis as a complementary feature could further enhance this approach by monitoring emotional states expressed in free-text entries alongside self-assessment scores such as PRISM-S. Future applications could use these data to identify periods of heightened distress or negative affect and trigger adaptive support strategies, including tailored prompts, coping suggestions, alerts to trusted contacts, low-effort just-in-time interventions during nighttime hours, and context-sensitive tasks aligned with app users’ daily rhythms. By combining circadian-aware engagement strategies with sentiment-informed personalization, digital tools could promote proactive use, strengthen resilience, and provide timely support when it is most needed. These suggestions should be considered as hypothesis-generating and not interpreted as evidence that such strategies would be effective, as this study does not evaluate intervention outcomes or causal mechanisms.

### Weekly and Seasonal Variations

The analysis showed temporal stability across the week in user engagement with the safety planning functionality, subjective perceived suffering (measured through PRISM-S), and sentiment expressed by free-text answers collected after the assessment. The lack of weekly cycles observed in this study suggests that the suicide prevention tool may reflect consistent usage patterns across the week, potentially protecting against typical day-of-week fluctuations in distress, help-seeking behavior, and social media posting about suicide, with elevated activity and risk often reported on Mondays in previous research [10,13-15,17]. However, no conclusions can be drawn about protective effects or the impact on fluctuations in distress or risk.

Since prior research reported stronger weekly technology-use rhythms in younger versus older individuals [38], the stable week-long activity observed in our study may indicate a more mature user population for the SERO app.

Future research could explore whether consistent daily use of safety planning as a digital tool helps reduce suicidal thoughts, behaviors, or high-risk social media activity, particularly during periods of elevated risk, such as Mondays. Studies could integrate ecological momentary assessment for finer-grained mood or ideation tracking, test interventions to boost nighttime engagement, and assess whether these temporal patterns are stable over time. These efforts could help examine whether and how engagement with the tool is associated with periods of heightened distress or risk.

The seasonal analysis of our data suggested modest fluctuations across the year in user interactions, perceived suffering, and sentiment expressed in free text. The number of interactions with the safety planning functionality was higher in autumn and lower in winter. At the same time, the sentiment expressed in free-text entries was more negative in autumn and least negative in winter. However, these differences were not statistically significant, indicating that app users engaged with the tool consistently across seasons. On the other hand, PRISM-S self-reported distances showed a small but significant seasonal pattern, with perceived suffering highest in winter and lowest in summer. These findings of lower perceived suffering in summer contrast with prior research reporting higher suicide rates during this period [15,20,21], highlighting that subjective perceptions of distress do not necessarily align with temporal patterns of suicidal behavior [9]. The better PRISM-S scores observed in winter contrast with research findings reporting higher risks around Christmas and New Year’s Day [14,18,21,22] and during spring [15,18-20].

These findings highlight the heterogeneity across populations, measures, and contexts. In previous research, objective indicators (eg, completed suicides or emergency presentations) have been measured. However, in this study, we used the PRISM-S tool, which is a subjective, visual-metaphorical measure of perceived proximity to suicidal crisis that indicates self-perceived suffering, and not necessarily lethal outcomes. This discrepancy highlights that subjective perceptions of distress and proximity to suicidality may diverge from the timing of suicidal behavior, as suicidal acts can sometimes occur after periods of heightened distress rather than during them. These patterns emphasize the need for incorporating tailored, season-aware suicide prevention strategies in interventions and assessment modalities (objective vs perceptual) to better address both temporal patterns in suicide outcomes reported in previous research and individuals’ lived experiences of distress.

### Study Limitations

This study has several limitations that should be considered when interpreting the findings. Users who chose to download and engage with the SERO app represent a self-selected population, likely already motivated to seek digital support for psychological distress or suicidal ideation. This introduces a substantial selection bias, as they may differ from nonusers in terms of help-seeking behavior, symptom severity, digital literacy, and access to health care. In addition, the app is recommended by health care professionals as part of therapy in some parts of Switzerland, which also represents a regional bias.

A major structural limitation is that the analyses rely on anonymized user logs from a single suicide prevention app, and it is unknown whether users belong to a clinical population, which limits the generalizability of the results to other populations, apps, or cultural and linguistic contexts, despite the app’s availability in multiple languages (German, French, Italian, and English). Additionally, the absence of detailed demographic or clinical information (eg, age, gender, diagnosis, concurrent treatment, or level of crisis severity) prevents subgroup analyses and limits exploration of heterogeneity in temporal usage patterns across different user types, such as individuals using the app preventively versus those experiencing acute distress. This study reflects real-world usage of the digital suicide prevention app without the ability to distinguish between users’ contextual needs and levels of psychological distress.

Another important limitation is the presence of unmeasured confounding factors that may influence observed temporal patterns. These could include clinical variables such as symptom severity, crisis context (preventive vs acute use), and concurrent treatment, as well as behavioral and technical factors such as intrauser repetition, heterogeneous usage intensity, device settings, and potential time zone differences. Because the dataset is fully anonymized and observational, these factors could not be measured or controlled for. Therefore, observed differences across the time of day, weekdays, or seasons should be interpreted as aggregated usage patterns rather than controlled within-person effects.

Additionally, the sentiment analysis was based on a pretrained multilingual transformer model without validation against human-coded data or clinically annotated benchmarks in this specific context. Therefore, the accuracy and reliability of affect classification should be interpreted with caution, particularly given the linguistic nuances and sensitivity of suicide-related content.

The relatively low frequency of PRISM-S use per user further limits the interpretation of temporal patterns. Many users completed the assessment only once or twice, which restricts the ability to capture within-person fluctuations over time. Additionally, app engagement may have decreased during periods of acute crisis due to reduced cognitive or emotional capacity, meaning that the absence of use at certain times (eg, nighttime) should not be interpreted as an absence of risk. Instead, usage patterns may better reflect moments when individuals have sufficient resources to seek support, rather than moments of highest suicidality.

A further limitation is the increased risk of type I error due to multiple statistical tests across temporal dimensions and outcomes. Although post hoc tests were adjusted within models, no global correction was applied. Therefore, statistically significant findings, particularly those with small effect sizes, should be interpreted as exploratory and hypothesis-generating rather than confirmatory.

It is important to note that engagement metrics serve as proxies for support needs but do not directly measure underlying suicidal ideation, distress levels, or clinical outcomes. Peaks in activity could reflect increased proactive coping, routine app use, notifications or reminders, or other external factors, reducing the specificity of inferences about temporal patterns in risk. For example, during the measurement period, 2 new app versions were released and communicated to the users, and the app was awarded an important prize in the Swiss health care system. Such events may have increased registrations or temporarily boosted activity independently of users’ psychological distress or support needs. Consequently, peaks in engagement may partly reflect product lifecycle effects, increased visibility, notifications, or renewed user interest rather than changes in suicidal ideation or clinical risk. In addition, uneven long-term retention may bias temporal analyses toward short-term or sporadic users, potentially underrepresenting sustained engagement patterns or dropout-related effects.

Due to the observational nature of the data, causal interpretations, conclusions about clinical effectiveness, risk reduction, or the impact of specific usage patterns on suicidal behavior are not possible. Observed temporal variations in engagement and sentiment cannot be definitively attributed to circadian, weekly, or seasonal influences on mental health without controlling for potential confounders such as user time zone inconsistencies, device usage habits, app updates, or other events during the study period (November 2022 to December 2025). Future research incorporating longitudinal designs, additional data sources, or multiple app platforms could strengthen the validity and generalizability of conclusions regarding temporal engagement and emotional patterns in digital suicide prevention tools, including designs that allow within-person analysis and adjustment for contextual and clinical covariates.

### Conclusions

This study highlights temporal patterns in the use of the suicide prevention app SERO. App users engaged most with safety planning during daytime hours, while perceived suffering (PRISM-S assessment) remained relatively stable but showed a subtle decline at night. Sentiment analysis of free-text responses from app users corroborated this pattern, showing more negative affective expressions during nighttime hours, particularly when perceived suffering was lower. Engagement was consistent across days of the week and largely unaffected by seasons, though small but nonsignificant seasonal fluctuations were observed in interactions, while perceived suffering and sentiment showed modest seasonal variation. These findings suggest that digital tools can support steady, routine-like coping, but periods of increased reported distress, especially at night, may be underaddressed, which should be interpreted separately from the timing of suicidal behavior. Given the observational design nature of this study, these findings should be interpreted as exploratory and hypothesis-generating rather than as evidence for intervention strategies. Future research could explore personalized, time-adaptive interventions that combine self-assessment and sentiment monitoring to enhance proactive engagement and provide timely support during periods of increased distress or need for support.

## Supplementary material

10.2196/95374 Multimedia Appendix 1 Safety plan interactions and mean PRISM-S (Pictorial Representation of Illness and Self-Measure—Suicidality) distance scores across morning, afternoon, and night.

10.2196/95374 Multimedia Appendix 2 Average interactions per safety planning topic and mean PRISM-S (Pictorial Representation of Illness and Self-Measure—Suicidality) distance across days of the week.

10.2196/95374 Checklist 1 STROBE checklist.

## Abbreviations

HSD: honestly significant difference
PRISM-S: Pictorial Representation of Illness and Self-Measure—Suicidality
SERO: Suicide Prevention: a Uniform Effort, Resource-Oriented
STROBE: Strengthening the Reporting of Observational Studies in Epidemiology
