# Supplementary material for: Temporal Patterns of Engagement and Sentiment in a Suicide Prevention Mobile App: Three-Year Observational Study
Source: JMIR Ment Health. 2026 Jul 16;13:e95374. doi: 10.2196/95374 (PMC13375210; doi:10.2196/95374)
Supplement: Multimedia Appendix 1 [file mental-v13-e95374-s001.docx]

**APPENDIX 1**

| 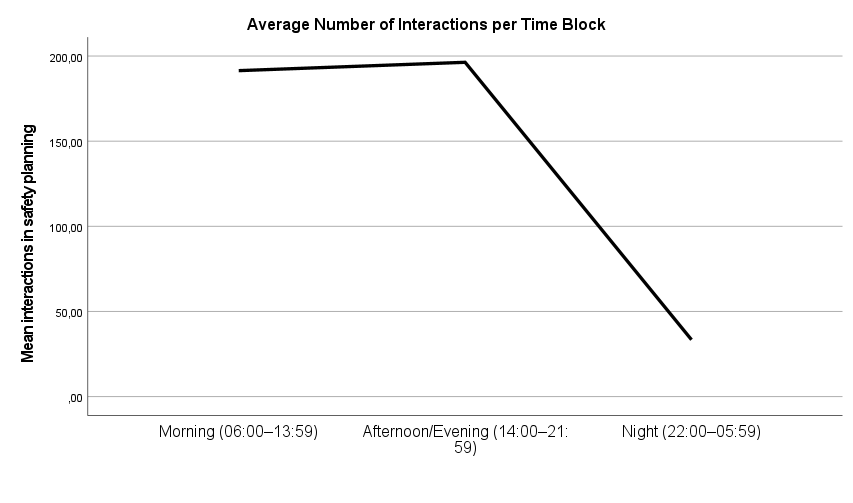  Figure A. Safety plan interactions across morning, afternoon and night | 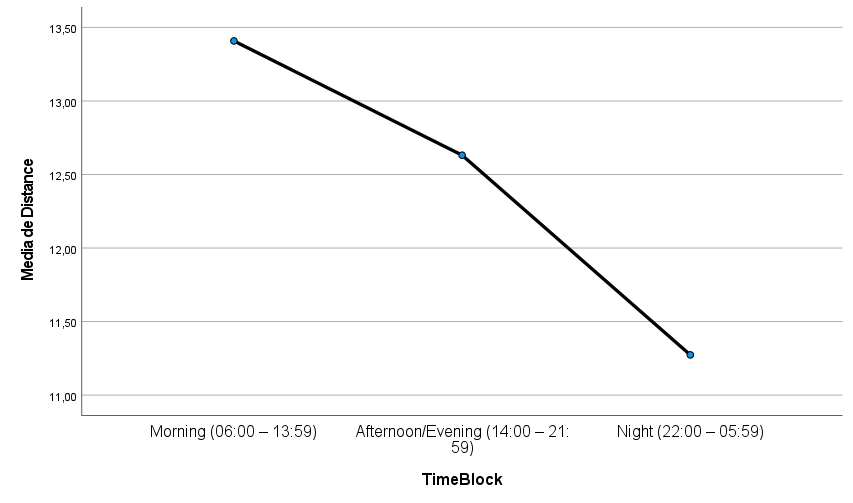  Figure B. Mean PRISM™-S distance scores across morning, afternoon and night |
| --- | --- |
