# Supplementary material for: Temporal Patterns of Engagement and Sentiment in a Suicide Prevention Mobile App: Three-Year Observational Study
Source: JMIR Ment Health. 2026 Jul 16;13:e95374. doi: 10.2196/95374 (PMC13375210; doi:10.2196/95374)
Supplement: Multimedia Appendix 2 [file mental-v13-e95374-s002.docx]

**APPENDIX 2**

| 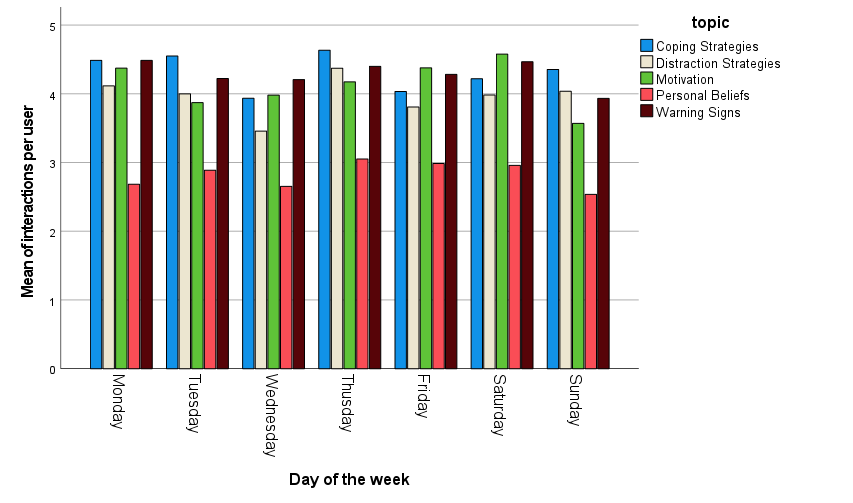Figure C. Average interactions per Safety planning topic across days of the week | 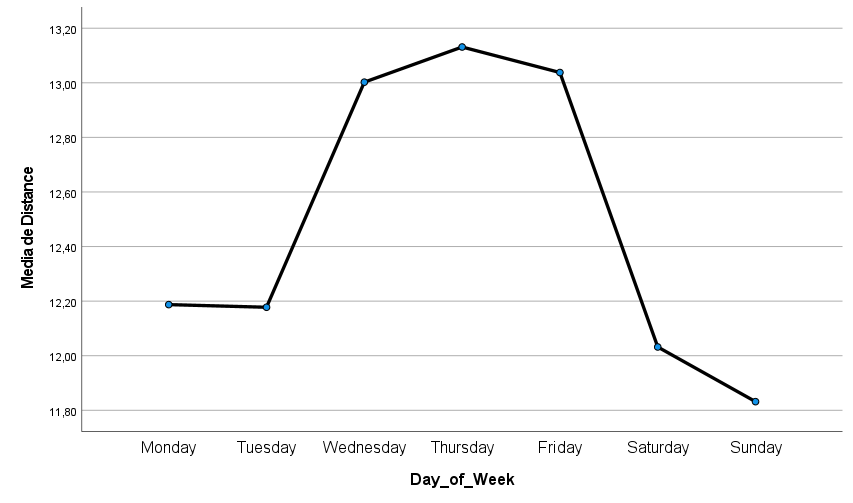  Figure D. Mean PRISM™-S distance scores across days of the week |
| --- | --- |
